# The relationship between hematocrit and serum albumin levels difference and mortality in elderly sepsis patients in intensive care units—a retrospective study based on two large database

**DOI:** 10.1186/s12879-022-07609-7

**Published:** 2022-07-18

**Authors:** Zichen Wang, Luming Zhang, Shaojin Li, Fengshuo Xu, Didi Han, Hao Wang, Tao Huang, Haiyan Yin, Jun Lyu

**Affiliations:** 1 Department of Intensive Care Unit, The First Affiliated Hospital of Jinan University, Guangzhou, Guangdong 510630 People’s Republic of China; 2 Department of Clinical Research, The First Affiliated Hospital of Jinan University, Guangzhou, Guangdong China; 3 Department of Public Health, University of California, Irvine, CA USA; 4 Department of Orthopaedics, The First Affiliated Hospital of Jinan University, Guangzhou, Guangdong 510630 People’s Republic of China; 5 Department of Statistics, Iowa State University, Ames, USA; 6 Guangdong Provincial Key Laboratory of Traditional Chinese Medicine Informatization, Guangzhou, Guangdong China

**Keywords:** Sepsis, Elderly, HCT-ALB, Mortality, Multi-center

## Abstract

**Background:**

Sepsis still threatens the lives of more than 300 million patients annually and elderly patients with sepsis usually have a more complicated condition and a worse prognosis. Existing studies have shown that both Hematocrit (HCT) and albumin (ALB) can be used as potential predictors of sepsis, and their difference HCT-ALB has a significant capacity to diagnose infectious diseases. Currently, there is no relevant research on the relationship between HCT-ALB and the prognosis of elderly sepsis patients. Therefore, this study aims to explore the association between HCT-ALB and mortality in elderly patients with sepsis.

**Methods:**

This study was a multi-center retrospective study based on the Medical Information Mart for Intensive Care (MIMIC-IV) database and the eICU Collaborative Research Database (eICU-CRD) in elderly patients with sepsis. The optimal HCT-ALB cut-off point for ICU mortality was calculated by the Youden Index based on the eICU-CRD dataset, and multivariate logistic regressions were conducted to explore the association between HCT-ALB and ICU/hospital mortality in the two databases. Subgroup analyses were performed for different parameters and comorbidity status.

**Results:**

The number of 16,127 and 3043 elderly sepsis patients were selected from two large intensive care databases (eICU-CRD and MIMIC-IV, respectively) in this study. Depending on the optimal cut-off point, patients in both eICU-CRD and MIMIC-IV were independently divided into low HCT-ALB (< 6.7) and high HCT-ALB (≥ 6.7) groups. The odds ratio (95%confidence interval) [OR (95CI%)] of the high HCT-ALB group were 1.50 (1.36,1.65) and 1.71 (1.58,1.87) for ICU and hospital mortality in the eICU-CRD database after multivariable adjustment. Similar trends in the ICU and hospital mortality [OR (95%CI) 1.41 (1.15,1.72) and 1.27 (1.07,1.51)] were observed in MIMIC-IV database. Subgroup analysis showed an interaction effect with SOFA score in the eICU-CRD database however not in MIMIC-IV dataset.

**Conclusions:**

High HCT-ALB (≥ 6.7) is associated with 1.41 and 1.27 times ICU and hospital mortality risk in elderly patients with sepsis. HCT-ALB is simple and easy to obtain and is a promising clinical predictor of early risk stratification for elderly sepsis patients in ICU.

**Supplementary Information:**

The online version contains supplementary material available at 10.1186/s12879-022-07609-7.

## Introduction

Sepsis is defined as life-threatening organ dysfunction caused by a dysregulated host response to infection, which is one of the most common complications in intensive-care unit (ICU) cases [1]. In clinical practice, organ dysfunction can be indicated by an increase of 2 or more points in the Sequential Organ Failure Assessment (SOFA) score [2, 3] Although the awareness of sepsis and medical technology have improved significantly recently, the number of sepsis patients each year still exceeds 30 million worldwide, and the total mortality rate is 17% [4], creating a high global disease burden. Sepsis is very common in elderly patients, with more than 50% of sepsis patients in the United States being over 65 years old [5]. Due to advanced age, immune system aging [6, 7], prolonged host inflammatory response time [8], and other factors, elderly patients have a more complicated disease occurrence and development and relatively difficult clinical treatment, making their mortality rate higher than that of nonelderly sepsis patients [9].

Synthesized in the liver, albumin (ALB) is the most abundant plasma protein, accounting for 40–60% of total plasma protein. ALB has various physiological functions, including expanding blood volume, maintaining plasma colloidal osmotic pressure, and providing nutrition and transportation. Serum ALB measurements are standard predictors in the prognosis of various diseases [10]. Hypoalbuminemia is common in critically ill patients and often causes a poor prognosis, with previous studies suggesting a correlation between serum ALB level and sepsis patient prognosis [11, 12]. Hematocrit (HCT) is the volume percentage of red blood cells. HCT can be increased by massive fluid loss leading to hemoconcentration and by the presence of chronic hypoxia in the patient. This relationship can also be observed in hemorrhagic fever, which is caused by certain viruses [13]. Several published studies have shown that HCT is a variable that can predict the risk of death in patients with sepsis and septic shock [14, 15]. The HCT and plasma ALB levels of healthy people are stable under normal conditions, and their values are in the ranges of 40–45% and 35–45 g/l [16], respectively. Sepsis can be caused by any type of infection, and it is common in conditions such as pneumonia, peritonitis, cholangitis, urinary system infection, cellulitis, meningitis, and abscesses. Its pathogenic microorganisms include bacteria, fungi, viruses, and parasites [17]. Infectious factors can activate the monocyte-macrophage system and other inflammatory response cells to produce and release many inflammatory mediators, including vasoactive substances, cytokines, chemokines, oxygen free radicals, and acute-phase reaction substances. Extensive damage to certain body systems and organs and increased capillary permeability leads to leakage of ALB and HCT fluctuations, altering their proportions [18]. In addition to the above effects of infection, the patient’s hypermetabolic state causes increased albumin consumption, intestinal dysfunction causes increased albumin loss, and patients with chronic hypoxic diseases can cause an increase in HCT, as well as blood loss and anemia, which are all factors that cause changes in HCT-ALB. The numerical difference value between HCT (%) and ALB (g/L) levels (HCT-ALB) is highly sensitive and specific among patients with infectious diseases and is, therefore, a potential indicator for the differential diagnosis of infections [19]. The relationship between HCT-ALB and the prognosis of elderly sepsis patients remains unclear. Therefore, the purpose of this study is to investigate the relationship between HCT-ALB and the prognosis of elderly patients with sepsis, and to provide a basis for clinical decision-making.

## Method

### Data source

All study data were extracted from the Medical Information Mart for Intensive Care-IV (MIMIC-IV) database and the eICU Collaborative Research Database (eICU-CRD). The MIMIC database is derived from the electronic medical records of the Beth Israel Deaconess Medical Center and includes data of basic demographics, ICU monitoring records, laboratory test results, and treatment prescriptions [20, 21]. MIMIC-IV version 0.4 was released in 2020 and includes over 250,000 case admissions from 2008 to 2019. eICU-CRD contains the electronic medical records of more than 200,000 patients admitted to the ICU of 208 hospitals in the United States in 2014 and 2015 [22]. Both databases were IRB pre-approved and de-identified databases under the requirements of the United States Health Insurance Portability and Accountability Act (HIPAA) to ensure the anonymity of patients’ personal information so informed consent is not necessary. All data were extracted by structured query language (SQL) Server.

### Study population

**Fig. 1:**
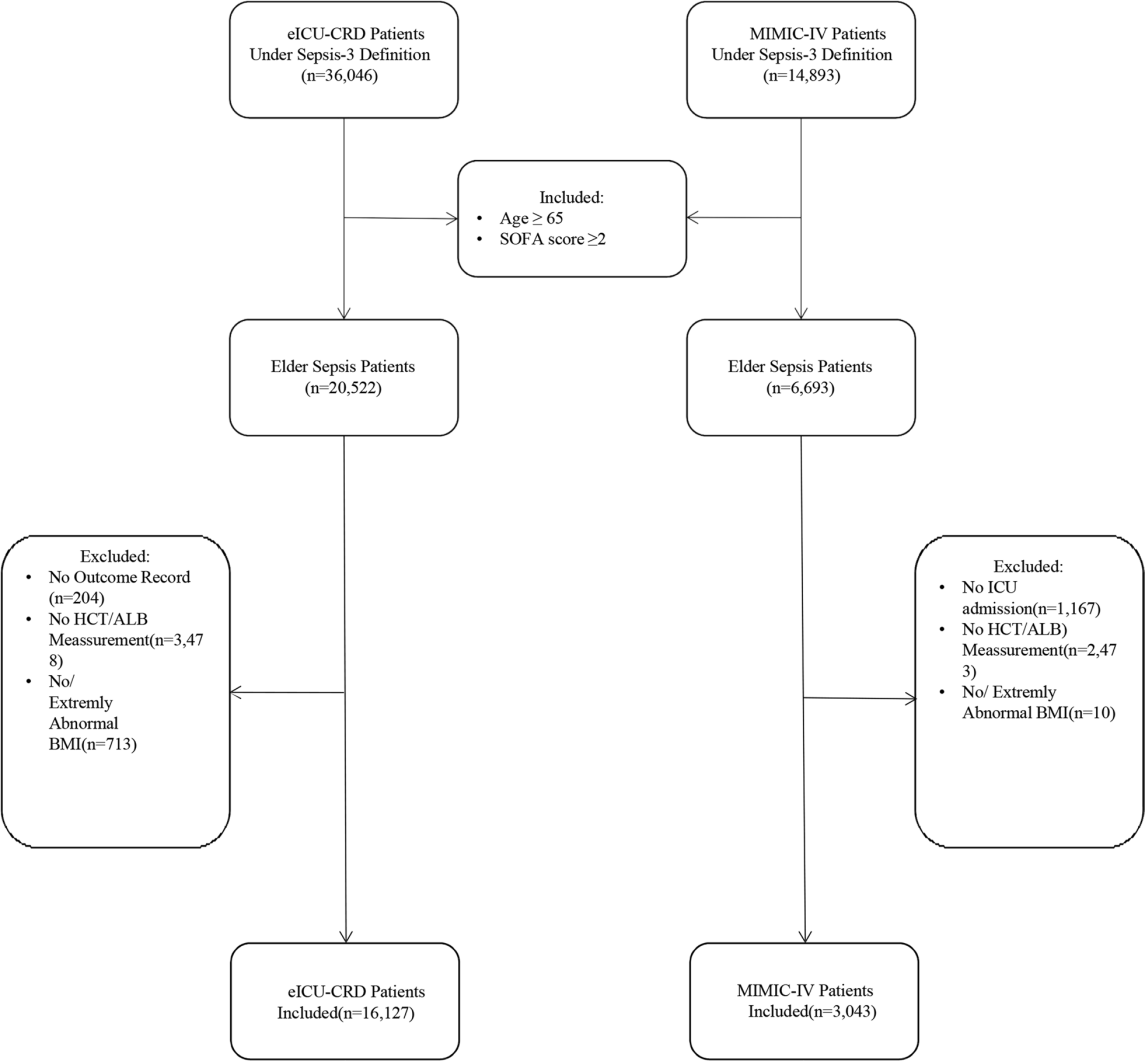
Inclusion and Exclusion Flowchart of the Study

The code to derive the SOFA score of MIMIC-IV and eICU-CRD was sourced from MIT Laboratory for Computational Physiology (https://github.com/MIT-LCP), which referenced the SOFA definition of previous researches [21, 22]. The number of elderly patients (age > 65 years) with sepsis meeting the Sepsis-3 definition (infected and with SOFA ≥ 2) [3] in the MIMIC-IV and eICU-CRD databases were 20,522 and 6693, respectively. Patients not admitted to ICU, without discharge status, without HCT-ALB records, and without BMI values or with an extremely abnormal BMI [BMI is associated with mortality in sepsis patients and is suggested to be adjusted for baseline characteristics [23]. However, as retrospective databases across multi-center, MIMIC-IV and eICU-CRD may have data entry errors and ununiformed units which may induce extremely abnormal BMI. To avoid potential bias, we removed patients with a BMI greater than 100.] were excluded, leaving 16,127 and 3043 patients for the study from the eICU-CRD database and MIMIC-IV for further analysis, respectively (Fig. 1).

### Outcomes and covariates

The primary outcome of this study was ICU mortality, and the secondary outcome was hospital mortality. The included variables were age, sex, HCT-ALB, BMI, SOFA score, ventilation use, dialysis use, vasopressor use, comorbidities: congestive heart failure (CHF), chronic pulmonary disease, diabetes, renal disease, malignant cancer, liver disease, and metastatic solid tumor. The HCT and ALB were extracted at the first record after ICU admission. BMI were grouped into underweight (BMI < 18.5 kg/m^2^), normal weight (18.5–24.9 kg/m^2^), overweight (25.0–29.9 kg/m^2^), and obese (≥ 30.0 kg/m^2^) [24].

### Statistical analysis

**Fig. 2:**
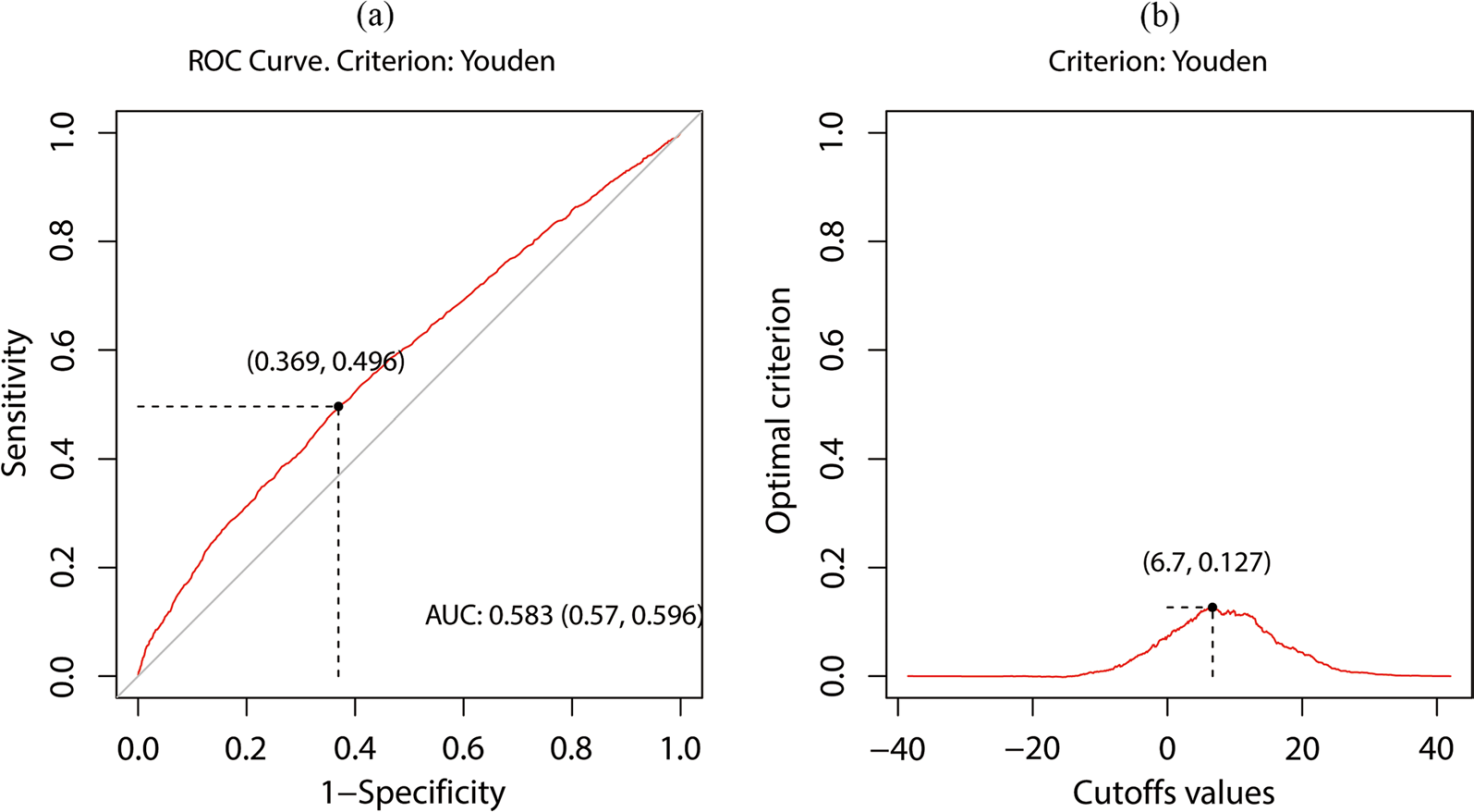
The result of optimal cut-off value calculated by Youden Index. **a** Receiver operating characteristic (ROC) curve for HCT-ALB. The black point represented the position of optimal point in ROC curve where sensitivity was 0.496 and specificity was 0.631. **b** The relationship between HCT-ALB and the position of max Youden Index. The black point represented the position of optimal cut-off value and the related Youden Index. The optimal cut-off point of HCT-ALB was 6.7; The max Youden Index was 0.127

The optimal HCT-ALB cut-off point for ICU mortality was calculated by the Youden Index of ROC curve based on the eICU-CRD dataset (Fig. 2) [25]. Depending on the optimal cut-off point (6.7), patients in both eICU-CRD and MIMIC-IV were independently divided into low HCT-ALB and high HCT-ALB groups. The median and interquartile range (IQR), counts, and percentages, described continuous and categorical variables, respectively. Kruskal–Wallis tests were conducted to analyze the differences between HCT-ALB groups in continuous variables, and chi-square tests were conducted to compare the categorical variables. Univariate and multivariate (adjusted by age, sex, HCT-ALB, BMI, SOFA score, ventilation use, dialysis use, vasopressor use, and comorbidities as potential confounders) logistic regressions were conducted to investigate the raw and adjusted between HCT-ALB groups and ICU/hospital mortality. Datasets of eICU-CRD and MIMIC-IV were analyzed individually to test the consistency of results in different populations.

To further exam the stability of results, subgroup analyses were performed in subgroups including Age (< 65 and ≥ 65 years), gender (male and female), SOFA score (< 6 and ≥ 6), BMI (underweight, normal weight, overweight, and obese) and comorbidities (no, yes) and tested the interaction effects.

A two-sided P value of < 0.05 was considered statistically significant. All data were analyzed using the R statistical software.

## Result

### Baseline clinical characteristics

In eICU-CRD dataset, the optimal cut-off point of HCT-ALB was 6.7 with a max Youden Index equal to 0.127. The confusion matrix for HCT, ALB, and HCT-ALB groups with optimal cut-off points was shown in (Additional file 1: Table S1). Compared to the confusion matrix of HCT and ALB, HCT-ALB was more balanced in sensitivity and specificity and had the highest balanced accuracy. The baseline characteristics of the study population were listed in (Table. 1). A total of 6238 (38.7%) eICU-CRD patients and 1073 (35.3%) MIMIC-IV patients were divided into high HCT-ALB groups. The median (IQR) of age in eICU-CRD and MIMIC-IV were 76 (70, 83) and 75 (69,82). The HCT level, ALB level, SOFA score, ventilation use, vasopressor use, CHF status, diabetes status, renal disease status, ICU mortality, and hospital mortality were significantly different between the HCT-ALB groups in the two datasets. Patients in high HCT-ALB groups had significantly higher HCT and lower ALB levels in all datasets. In addition, the difference in gender, BMI groups, dialysis use, liver disease status, and metastatic solid tumor status between HCT-ALB groups were significant for the eICU-CRD dataset while age and malignant cancer status were significant for the MIMIC-IV dataset.

**Table 1:** The Baseline clinical characteristics of eICU-CRD and MIMIC-IV patients

	eICU-CRD				MIMIC-IV			
	Total (n = 16127)	HCT-ALB < 6.7 (n = 9899)	HCT-ALB ≥ 6.7 (n = 6238)	P value	Total (n = 3043)	HCT-ALB < 6.7 (n = 1970)	HCT-ALB ≥ 6.7 (n = 1073)	P value
Age	76 (70,83)	76 (70,83)	76 (70,83)	0.532	75 (69,82)	75 (69, 81)	76 (70, 82)	0.048
HCT (%)	30.8 (27.0,35.4)	28. (25.7,32.6)	34.3 (30.4,38.8)	< 0.001	32.0 (27.6,36.3)	29.7 (25.9, 33.5)	36.3 (32.9, 40.1)	< 0.001
ALB (g/L)	27.0 (22.0,32.0)	29.0 (25.0,34.0)	22.0 (19.0,26.0)	< 0.001	28.0 (24.0,32.0)	30.0 (26.0, 34.0)	24.0 (21.0, 28.0)	< 0.001
SOFA	7 (5,9)	7 (5,9)	7 (5,9)	< 0.001	7 (5,10)	7 (4, 10)	8 (5, 11)	< 0.001
Gender (%)				0.010				0.445
Male	8607 (53.4)	5198 (52.6)	3409 (54.6)		1686 (55.4)	1102 (55.9)	584 (54.4)	
Female	7520 (46.6)	4691 (47.4)	2829 (45.4)		1357 (44.6)	868 (44.1)	489 (45.6)	
BMI (%)				< 0.001				0.322
Underweight	828 (5.1)	449 (4.5)	379 (6.1)		154 (5.1)	89 (4.5)	65 (6.1)	
Normalweight	5110 (31.7)	3022 (30.6)	2088 (33.5)		965 (31.7)	628 (31.9)	337 (31.4)	
Overweight	4520 (28.0)	2843 (28.7)	1677 (26.9)		876 (28.8)	573 (29.1)	303 (28.2)	
Obese	5669 (35.2)	3575 (36.2)	2094 (33.6)		1048 (34.4)	680 (34.5)	368 (34.3)	
Ventilation (%)				< 0.001				0.001
No	11,202 (69.5)	7061 (71.4)	4141 (66.4)		356 (11.7)	259 (13.1)	97 (9.0)	
Yes	4925 (30.5)	2828 (28.6)	2097 (33.6)		2687 (88.3)	1711 (86.9)	976 (91.0)	
Dialysis (%)				< 0.001				0.712
No	14,389 (89.7)	8656 (87.4)	5743 (92.1)		2724 (89.5)	1760 (89.3)	964 (89.8)	
Yes	1738 (10.3)	1243 (12.6)	495 (7.9)		319 (10.5)	210 (10.7)	109 (10.2)	
Vasopressor (%)				< 0.001				< 0.001
No	11,271 (69.9)	7205 (72.8)	4066 (65.2)		1218 (40.0)	859 (43.6)	359 (33.5)	
Yes	4856 (30.1)	2684 (27.2)	2172 (34.8)		1825 (60.0)	1111 (56.4)	714 (66.5)	
Congestive heart Failure				< 0.001				0.040
No	12,162 (75.4)	7310 (73.9)	4852 (77.8)		1712 (56.3)	1081 (54.9)	631 (58.8)	
Yes	3965 (24.6)	2579 (26.1)	1386 (22.2)		1331 (43.7)	889 (45.1)	442 (41.2)	
Chronic pulmonary disease (%)				0.941				0.450
No	12,804 (79.4)	7849 (79.4)	4955 (79.4)		2108 (69.3)	1355 (68.8)	753 (70.2)	
Yes	3323 (20.6)	2040 (20.6)	1283 (20.6)		935 (30.7)	615 (31.2)	320 (29.8)	
Diabetes (%)				< 0.001				0.001
No	13,409 (83.1)	8097 (81.9)	5312 (85.2)		1923 (63.2)	1204 (61.1)	719 (67.0)	
Yes	2718 (16.9)	1792 (18.1)	926 (14.8)		1120 (36.8)	766 (38.9)	354 (33.0)	
Renal disease (%)				< 0.001				< 0.001
No	14,669 (91.0)	8867 (89.7)	5802 (93.0)		2025 (66.5)	1240 (62.9)	785 (73.2)	
Yes	1458 (9.0)	1022 (10.3)	436 (7.0)		1018 (33.5)	730 (37.1)	288 (26.8)	
Malignant cancer (%)				0.314				0.006
No	12,699 (78.7)	7761 (78.5)	4938 (79.2)		2428 (79.8)	1542 (78.3)	886 (82.6)	
Yes	3428 (21.3)	2128 (21.5)	1300 (20.8)		615 (20.2)	428 (21.7)	187 (17.4)	
Liver disease (%)				< 0.001				0.271
No	15,352 (95.2)	9,461 (95.7)	5891 (94.4)		2586 (85.0)	1685 (85.5)	901 (84.0)	
Yes	775 (4.8)	428 (4.3)	347 (5.6)		457 (15.0)	285 (14.5)	172 (16.0)	
Metastatic solid tumor (%)				0.069				0.247
No	15,591 (96.7)	9,581 (96.9)	6010 (96.3)		2779 (91.3)	1790 (90.9)	989 (92.2)	
Yes	536 (3.3)	308 (3.1)	228 (3.7)		264 (8.7)	180 (9.1)	84 (7.8)	
ICU mortality (%)				< 0.001				< 0.001
No	13,899 (86.2)	8766 (88.6)	5133 (82.3)		2377 (78.1)	1601 (81.3)	776 (72.3)	
Yes	2228 (13.8)	1123 (11.4)	1105 (17.7)		666 (21.9)	369 (18.7)	297 (27.7)	
Hospital mortality (%)				< 0.001				< 0.001
No	12,585 (78.0)	8128 (82.2)	4457 (71.4)		2065 (67.9)	1391 (70.6)	674 (62.8)	
Yes	3542 (22.0)	1761 (17.8)	1781 (28.6)		978 (32.1)	579 (29.4)	399 (37.2)	

Continuous variables were expressed by median (interquartile), categorical variables were expressed by count (percentage)

P values were calculated to compare difference between HCT-ALB groups for each dataset separately

### Logistic regression

(Table. 2) showed that compared to the reference group, the high HCT-ALB group had increased univariate ICU and hospital mortality risk [OR (95%CI) = 1.68 (1.54,1.84) and 1.84 (1.71,1.99)] in the eICU- CRD datasets. After adjusted by co-variables, the OR (95%CI) for ICU and hospital mortality for the eICU-CRD population were 1.50 (1.36,1.65) and 1.71 (1.58,1.87), demonstrating that high HCT-ALB was independently associated and had 42% and 66% elevated risk of ICU and hospital mortality, respectively. The results of the MIMIC-IV data set also showed high consistency with eICU-CRD. The univariate regressions result revealed that high HCT-ALB was also positively correlated to ICU and hospital mortality [OR (95%CI) = 1.66 (1.39,1.98) and 1.42 (1.22,1.66)] in MIMIC-IV dataset. After multivariate adjustment, the OR (95%CI) for MIMIC-IV population were 1.41 (1.15,1.72) and 1.27 (1.07,1.51), respectively. MIMIC-IV result showed that higher HCT-ALB group, divided by the cut-off value from eICU-CRD dataset, was also independently had a 41% and 26% higher risk of ICU and hospital mortality in external dataset. The results of all co-variables were showed in (Additional file 1: Tables S2,3).

**Table 2:** Analysis for association between HCT-ALB group and outcomes

	ICU mortality		Hospital mortality	
	OR (95% CI)	P value	OR (95% CI)	P value
eICU-CRD				
Univariable model	1.68 (1.54, 1.84)	< 0.001	1.84 (1.71, 1.99)	< 0.001
Multivariable model	1.50 (1.36, 1.65)	< 0.001	1.71 (1.58, 1.87)	< 0.001
MIMIC-IV				
Univariable model	1.66 (1.39, 1.98)	< 0.001	1.42 (1.22, 1.66)	< 0.001
Multivariable model	1.41 (1.15, 1.72)	0.001	1.27 (1.07, 1.51)	0.007

Multivariate models were adjusted by Age, Gender, BMI group, SOFA score, Ventilation, Dialysis, Vasopressor use, Comorbidities (CHF, Chronic Pulmonary Disease, Diabetes, Renal disease, Malignant cancer, Liver disease, and Metastatic solid tumor), excluding models belonging subgroup variate

*OR* odds ratio, *CI* confidence interval

The OR (95CI%) were calculated when low HCT-ALB (< 6.7) groups set as reference

### Subgroup analysis

The subgroup analyses revealed the association between HCT-ALB and outcomes under different statuses in eICU-CRD and MIMIC-IV (Table. 3) (Table. 4). In eICU-CRD dataset, ORs for ICU mortality were not statistically significant for patients with normalweight, with liver disease, and with metastatic solid tumor. In the MIMIC-IV dataset, the association between the high HCT-ALB group and ICU/hospital mortality was not significant in subgroups including patients over 80, male, underweight/obese, without CHF, without diabetes, with chronic pulmonary disease, with malignant cancer, and with metastatic solid tumor. No meaning ORs for hospital mortality were also observed in overweight, without renal disease, and with liver disease. However, the only significant interaction effects were SOFA scores for hospital mortality (P = 0.040) in eICU-CRD while no interaction effect for any subgroup was observed in the MIMIC-IV dataset. In summary, the association between HCT-ALB and outcomes was consistently positive in different subgroups.

**Table 3:** Subgroup analysis for association between HCT-ALB group and outcomes for eICU-CRD

		ICU mortality			Hospital mortality		
Subgroups	N	OR (95%CI)	P value	P—interaction	OR (95%CI)	P value	P—interaction
Total	16,127	1.50 (1.36, 1.65)	< 0.001	/	1.71 (1.58, 1.87)	< 0.001	/
Age				0.390			0.861
65–80	10,624	1.60 (1.41, 1.80)	< 0.001		1.76 (1.59, 1.96)	< 0.001	
> 80	5503	1.43 (1.22, 1.69)	< 0.001		1.73 (1.52, 1.98)	< 0.001	
Gender				0.353			0.208
Male	8607	1.43 (1.25, 1.64)	< 0.001		1.63 (1.46, 1.83)	< 0.001	
Female	7052	1.57 (1.36, 1.82)	< 0.001		1.82 (1.62, 2.06)	< 0.001	
SOFA				0.064			0.040
< 6	5592	1.65 (1.29, 2.11)	< 0.001		1.89 (1.59, 2.26)	< 0.001	
≥ 6	10,535	1.50 (1.30, 1.67)	< 0.001		1.70 (1.55, 1.68)	< 0.001	
BMI				0.324			0.658
Underweight	828	1.93 (1.29, 2.91)	0.001		1.86 (1.35, 2.61)	< 0.001	
Normalweight	5110	1.55 (0.68, 3.60)	0.299		2.00 (1.13, 3.56)	0.017	
Overweight	4520	1.78 (0.95, 3.35)	0.007		2.32 (1.51, 3.95)	< 0.001	
Obese	5669	1.91 (1.20, 3.02)	0.006		2.01 (1.45, 2.78)	< 0.001	
Congestive heart failure				0.809			0.499
No	12,162	1.48 (1.32, 1.66)	< 0.001		1.69 (1.54, 1.86)	< 0.001	
Yes	3965	1.54 (1.27, 1.88)	< 0.001		1.81 (1.54, 2.14)	< 0.001	
Chronic pulmonary disease				0.753			0.262
No	12,804	1.49 (1.33, 1.69)	< 0.001		1.68 (1.53, 1.85)	< 0.001	
Yes	3323	1.56 (1.26, 1.94)	< 0.001		1.87 (1.56, 2.23)	< 0.001	
Diabetes				0.577			0.621
No	13,409	1.54 (1.39, 1.72)	< 0.001		1.73 (1.53, 1.89)	< 0.001	
Yes	2718	1.66 (1.33, 2.05)	< 0.001		1.72 (1.38, 2.13)	< 0.001	
Renal disease				0.387			0.206
No	14,669	1.49 (1.34, 1.65)	< 0.001		1.70 (1.56, 1.85)	< 0.001	
Yes	1458	1.65 (1.17, 2.32)	< 0.001		1.99 (1.50, 2.66)	< 0.001	
Malignant cancer				0.984			0.721
No	12,699	1.49 (1.34, 1.67)	< 0.001		1.71 (1.55, 1.88)	< 0.001	
Yes	3428	1.47 (1.21, 1.81)	< 0.001		1.76 (1.49, 2.09)	< 0.001	
Liver disease				0.735			0.435
No	15,352	1.50 (1.36, 1.67)	< 0.001		1.71 (1.57, 1.86)	< 0.001	
Yes	775	1.39 (0.97, 2.01)	0.073		1.99 (1.44, 1.78)	< 0.001	
Metastatic solid tumor				0.953			0.324
No	15,591	1.50 (1.35, 1.66)	< 0.001		1.70 (1.57,1.86)	< 0.001	
Yes	536	1.51 (0.92, 2.48)	0.104		2.21 (1.47, 3.34)	< 0.001	

Multivariate models were adjusted by Age, Gender, BMI group, SOFA score, Ventilation, Dialysis, Vasopressor use, Comorbidities (CHF, Chronic Pulmonary Disease, Diabetes, Renal disease, Malignant cancer, Liver disease, and Metastatic solid tumor), excluding models belonging subgroup variate

BMI subgroup were defined as underweight (BMI < 18.5 kg/m^2^), normal weight (18.5–24.9 kg/m^2^), overweight (25.0–29.9 kg/m^2^), and obese (≥ 30.0 kg/m^2^)

*OR* odds ratio, *CI* confidence interval

The OR (95CI%) were calculated when low HCT-ALB (< 6.7) groups set as reference

**Table 4:** Subgroup analysis for association between HCT-ALB group and outcomes for MIMIC-IV

		ICU mortality			Hospital mortality		
Subgroups	N	OR (95%CI)	P value	P—interaction	OR (95%CI)	P value	P—interaction
Total	3043	1.41 (1.15, 1.72)	0.001	/	1.26 (1.07, 1.51)	0.007	/
Age				0.707			0.438
65–80	2156	1.61 (1.27, 2.05)	< 0.001		1.43 (1.15, 1.746)	0.001	
> 80	887	1.14 (0.79, 1.64)	0.473		1.01 (0.73, 1.38)	0.964	
Gender				0.189			0.051
Male	1686	1.28 (0.97, 1.68)	0.075		1.09 (0.86, 1.38)	0.488	
Female	1357	1.64 (1.21, 2.22)	0.001		1.54 (1.20, 1.99)	0.001	
SOFA				0.433			0.446
< 6	994	2.37 (1.37, 4.10)	0.002		1.56 (1.07, 2.24)	0.018	
≥ 6	2049	1.39 (1.12, 1.71)	0.002		1.25 (1.03, 1.52)	0.022	
BMI				0.683			0.405
Underweight	154	1.03 (0.35, 2.94)	0.954		1.01 (0.42, 2.39)	0.986	
Normalweight	965	8.94 (1.55, 73.6)	0.021		2.72 (1.01, 7.25)	0.044	
Overweight	876	8.20 (1.51, 58.6)	0.018		2.32 (0.94, 5.69)	0.065	
Obese	1048	1.23 (0.43, 3.36)	0.689		0.84 (0.42, 1.61)	0.609	
Congestive heart failure				0.302			0.214
No	1712	1.25 (0.94, 1.65)	0.119		1.13 (0.89, 1.42)	0.324	
Yes	1331	1.62 (1.21, 2.18)	0.001		1.46 (1.12, 1.89)	0.005	
Chronic pulmonary disease				0.395			0.623
No	2108	1.49 (1.16, 1.91)	0.002		1.31 (1.06, 1.61)	0.013	
Yes	935	1.28 (0.90, 1.82)	0.162		1.20 (0.88, 1.64)	0.254	
Diabetes				0.226			0.136
No	1923	1.28 (0.99, 1.64)	0.056		1.14 (0.92, 1.42)	0.214	
Yes	1120	1.75 (1.24, 2.46)	0.001		1.54 (1.15, 2.07)	0.004	
Renal disease				0.401			0.269
No	2025	1.32 (1.03, 1.70)	0.027		1.16 (0.94, 1.45)	0.179	
Yes	1018	1.60 (1.13, 2.26)	0.008		1.47 (1.09, 1.98)	0.012	
Malignant cancer				0.758			0.072
No	2428	1.47 (1.17, 1.84)	< 0.001		1.39 (1.15, 1.69)	< 0.001	
Yes	615	1.41 (0.88, 2.24)	0.148		0.925 (0.61, 1.39)	0.713	
Liver disease				0.362			0.554
No	2586	1.39 (1.11, 1.73)	0.004		1.25 (1.03, 1.51)	0.027	
Yes	457	1.73 (1.05, 2.87)	0.031		1.46 (0.94, 2.26)	0.091	
Metastatic solid tumor				0.639			0.875
No	2779	1.44 (1.17, 1.79)	< 0.001		1.28 (1.06, 1.53)	0.009	
Yes	264	1.28 (0.65, 2.50)	0.464		1.36 (0.73, 2.52)	0.328	

Multivariate models were adjusted by Age, Gender, BMI group, SOFA score, Ventilation, Dialysis, Vasopressor use, Comorbidities (CHF, Chronic Pulmonary Disease, Diabetes, Renal disease, Malignant cancer, Liver disease, and Metastatic solid tumor), excluding models belonging subgroup variate

BMI subgroup were defined as underweight (BMI < 18.5 kg/m^2^), normal weight (18.5–24.9 kg/m^2^), overweight (25.0–29.9 kg/m^2^), and obese (≥ 30.0 kg/m^2^)

*OR* odds ratio, *CI* confidence interval

The OR (95CI%) were calculated when low HCT-ALB (< 6.7) groups set as reference

## Discussion

In this multi-center retrospective study based on two large public databases MIMIC-IV and eICU-CRD, HCT-ALB was related to the prognosis of elderly sepsis patient. We used the eICU-CRD database to calculate the optimal cut-off point and grouped patients from two datasets independently. Consistent results were obtained in both datasets, and the external dataset result demonstrated that when HCT-ALB was greater than 6.7, the risk of death in the ICU and in-hospital of elderly patients with sepsis will increase by about 1.41 and 1.27 times. Although previous studies had studied the relationship between HCT-ALB and infectious diseases/hypertension in pregnancy [26, 27], our research firstly explored the relationship between this indicator and the prognosis of elderly sepsis patients in the ICU. Elderly patients with sepsis have high mortality rates and are often delayed in diagnosis due to complications characteristics [28], weakened immunity, and atypical clinical manifestations [29]. Studies have shown that the mortality rate of sepsis patients increases with age, and the mortality rate of elderly patients (≥ 80 years old) is higher than that of patients 65–79 years old [30].

In critically ill patients, hypoproteinemia is a common phenomenon [31, 32]. It is firstly associated with patients being bedridden for long periods, in a state of fasting and drinking, with inadequate nutritional intake [33, 34]. When patients suffered from sepsis, the pathogenic microorganisms in the foci of infection and the various toxins released by them can stimulate the immune system of the body to release a large number of inflammatory mediators such as tumor necrosis factor and interleukins [35], which can impair the function of the vascular endothelial barrier, increase capillary permeability and cause a large amount of plasma extravasation, resulting in a decrease in blood volume and a decrease in the concentration of ALB in plasma [36]. Under normal conditions, the gap between capillary endothelial cells is 6–7 nm [37, 38], and the diameter of red blood cells is 7–8 μm (1000 times larger than the gap), meaning that red blood cells cannot pass through, and so maintaining HCT levels. However, patients with sepsis can experience a relative increase in HCT due to increased vascular permeability, which leads to massive extravasation of body fluids. In addition to these, the depletion produced by the hypermetabolic state of sepsis patients and the gastrointestinal dysfunction can lower the level of ALB [39]. When the patient's plasma albumin decreases due to the above-mentioned various factors, the plasma colloid osmotic pressure in turn will decrease, a large amount of liquid will be retained in the interstitial space [40], and the effective circulating blood volume will be further reduced, red blood cells will aggregate, blood viscosity will increase, and microcirculation disorders will be aggravated [41, 42]. It can lead to organ ischemia and focal necrosis, which can lead to organ failure and aggravate the condition of sepsis patients if it is severe or lasts for a long time [43]. Therefore, in elderly patients with sepsis, the HCT-ALB difference can reflect the degree of the patient's condition, and the corresponding therapeutic measures, such as albumin infusion and replenishment of effective circulating blood volume, should be taken according to the level of the difference to improve the patient's prognosis.

The results of subgroup analysis indicated that in the eICU-CRD dataset, in the group with HCT-ALB greater than 6.7, compared with people with SOFA ≥ 6, hospital death in patients with SOFA score < 6 is significantly higher, and there was an interaction, which was not observed in the MIMIC-IV database. Among patients with SOFA greater than 6 in the eICU-CRD dataset, the decrease of HCT may be due to the aggravation of the patient's condition, the increase of erythrocyte destruction and the change of plasma volume caused by the increase of fluid supplement, thus reducing the HCT-ALB difference [44, 45]. However, as the optimal value was calculated from the eICU-CRD dataset, this interaction effect may be biased which can also explain why similar result was not observed in external datasets. In addition, the heterogeneity of databases leads to different results, which is understandable. The ORs for mortality were not statistically significant for subgroups including BMI, liver disease, and metastatic solid tumor in eICU-CRD dataset while in the MIMIC-IV dataset, the association between the high HCT-ALB group and mortality was not significant in subgroups including age, gender, BMI, CHF, diabetes, chronic pulmonary disease, renal disease, malignant cancer, and metastatic solid tumor. The heterogeneity between databases is one of the reasons for this phenomenon, and secondly this may be due to the aging and physiological decline of the immune system in patients over 80 years of age [46, 47], hence the relatively small difference in HCT-ALB reflecting the level of inflammation.

## Strengths and limitations of the study

To the best of our knowledge, this is the first study on the relationship between HCT-ALB and mortality in elderly patients with sepsis in the ICU. HCT-ALB is a relatively easy-to-measure index, which can facilitate risk stratification and serve as a reference for early clinical intervention decisions. As a large-sample multi-center study, our results are representative, reliable, and extrapolative. Just as we segmented the HCT-ALB with the best threshold in the eICU-CRD, we got consistent results in the MIMIC-IV database. There are, of course, some limitations to this study. First, it is a retrospective study, and some confounding bias is inevitable. Second, the disease severity scoring system used in this study was the SOFA score, and the Acute Physiology and Chronic Health Evaluation (APACHE) II score may make the study more robust in the future when circumstances permit. Third, in this study, liver disease was adjusted as a covariate, and it might be better to exclude patients with liver dysfunction in future studies. Fourth, we did not assess the nutritional and anemia status of the patients. In addition, as an external validation dataset, the ICU/hospital mortality risks of the high HCT-ALB group in the MIMIC-IV dataset were lower than that of eICU-CRD. In the subgroup analysis, although only the SOFA subgroup in the eICU-CRD dataset showed a significant interaction, there was heterogeneity in the ORs observed in the eICU-CRD between MIMIC-IV subgroups, which also weakened the association of the HCT-ALB and mortality risks. Therefore, more studies are needed to further validate the interaction between subgroup analysis populations.

## Conclusions

High HCT-ALB (≥ 6.7) is associated with 1.41 and 1.27 times of ICU and hospital mortality risk in elderly patients with sepsis. HCT-ALB is simple and easy to obtain and is a promising clinical predictor of early risk stratification for elderly sepsis patients in ICU.

## Supplementary Information

**Additional file 1: Table S1. **The evaluation metrics of confusion matrix of three univariables. **Table S2**. The result of multivariate regressions of eICU-CRD dataset. **Table S3**. The result of multivariate regressions of MIMIC-IV dataset.

## Data Availability

The data were available on the MIMIC-IV website at https://mimic.physionet.org/,10.13026/C2HM2Qand eICU-CRD at. The data in this article can be reasonably applied to the corresponding author.

## Abbreviations

ICU: Intensive care units
HCT: Hematocrit
ALB: Albumin
HCT-ALB: Hematocrit albumin difference
MIMIC-IV: Medical Information Mart for Intensive Care-IV
eICU-CRD: EICU Collaborative Research Database
SOFA: Sequential Organ Failure Assessment
OR: Odds ratio
CI: Confidence interval
APACHE: Acute Physiology and Chronic Health Evaluation
