# Supplementary material for: The relationship between hematocrit and serum albumin levels difference and mortality in elderly sepsis patients in intensive care units—a retrospective study based on two large database
Source: BMC Infect Dis. 2022 Jul 18;22:629. doi: 10.1186/s12879-022-07609-7 (PMC9295343; doi:10.1186/s12879-022-07609-7)
Supplement: Supplementary file 1 — Additional file 1: Table S1. The evaluation metrics of confusion matrix of three univariables. Table S2. The result of multivariate regressions of eICU-CRD dataset. Table S3. The result of multivariate regressions of MIMIC-IV dataset. [file 12879_2022_7609_MOESM1_ESM.docx]

**Title Page**

**Title:** The Relationship between Hematocrit and Serum Albumin Levels Difference and Mortality in Elderly Septic Patients in Intensive Care Units – A Retrospective Study Based on Two Large Database

**The name(s) of the author(s):**, Zichen Wang, MPH^1,3a^, Luming Zhang, MD^1,2a^, Shaojin Li^4^, Fengshuo Xu, BD^2^, Didi Han, BD^2^, Hao Wang^5^,Tao Huang^2^, Haiyan Yin, MD, phD^1*^, Jun Lyu, MD, phD^2*^

^a^ Zichen Wang and Luming Zhang contributed equally to the study.

**The affiliation(s) and address(es) of the author(s):**

1. Intensive Care Unit, The First Affiliated Hospital of Jinan University, Guangzhou, Guangdong Province, China;
2. Department of Clinical Research, The First Affiliated Hospital of Jinan University, Guangzhou, Guangdong Province, China;
3. Department of Public Health, University of California, Irvine, United State;
4. Department of Orthopaedics, The First Affiliated Hospital of Jinan University, Guangzhou, Guangdong Province, China;
5. Department of Statistics, Iowa state university, Ames, United States;

**Corresponding author**: Dr. Jun Lyu; Dr. Haiyan Yin

**Address:** Department of Clinical Research, The First Affiliated Hospital of Jinan University, Guangzhou 510630, People’s Republic of China; Intensive Care Unit, The First Affiliated Hospital of Jinan University, Guangzhou 510630, People’s Republic of China

**Tel:** +86-13922274169; +86-13318831222

**e-mail:** [lyujun2020@jnu.edu.cn](mailto:lujun2006@xjtu.edu.cn); yinhaiyan1867@126.com

**ORCID:** 0000-0002-2237-8771

**Funding:** This work was supported by the National Natural Science Foundation of China (No. 82072232; 81871585), the Natural Science Foundation of Guangdong Province (No. 2018A030313058), Technology and Innovation Commission of Guangzhou Science, China (No.201804010308).

**Conflicts of interest:** The authors report no conflicts of interest in this work.

**Ethical approval:** All procedures performed in studies involving human participants were in accordance with the ethical standards of the institutional and national research committee and with the 1964 Helsinki declaration and its later amendments or comparable ethical standards.

| Variable | HCT | ALB | HCT-ALB |
| --- | --- | --- | --- |
| Optimal cut-off value | 36.4 | 24.5 | 6.7 |
| Sensitivity | 0.133 | 0.508 | 0.496 |
| Specificity | 0.893 | 0.357 | 0.631 |
| Accuracy | 0.788 | 0.378 | 0.612 |
| Balanced Accuracy | 0.513 | 0.433 | 0.563 |

### Additional file 1: Table S1 The evaluation metrics of confusion matrix of three univariables

|  | ICU Mortality P value | | Hospital Mortality P value | |
| --- | --- | --- | --- | --- |
| Variable | OR(95%CI) | P value | OR(95%CI) | P value |
| Age | 1.02(1.01,1.03) | <0.001 | 1.03(1.02,1.04) | <0.001 |
| SOFA | 1.20(1.18,1.22) | <0.001 | 1.18(1.16,1.20) | <0.001 |
| Gender |  |  |  |  |
| Male | Ref | | | |
| Female | 1.06(0.96,1.17) | 0.229 | 1.10(1.01,1.19) | 0.030 |
| BMI |  |  |  |  |
| Underweight | Ref | | | |
| Normalweight | 0.68(0.55,0.85) | <0.001 | 0.65(0.55,0.78) | <0.001 |
| Overweight | 0.63(0.51,0.78) | <0.001 | 0.57(0.48,0.69) | <0.001 |
| Obese | 0.58(0.47,0.72) | <0.001 | 0.51(0.42,0.61) | <0.001 |
| Ventilation |  |  |  |  |
| No | Ref | | | |
| Yes | 2.18(1.96,2.43) | <0.001 | 2.04(1.85,2.23) | <0.001 |
| Dialysis |  |  |  |  |
| No | Ref | | | |
| Yes | 1.27(1.10,1.48) | 0.001 | 1.22(1.06,1.39) | 0.005 |
| Vasopressor |  |  |  |  |
| No | Ref | | | |
| Yes | 2.16(1.95,2.39) | <0.001 | 1.84(1.68,2.00) | <0.001 |
| Congestive heart Failure |  |  |  |  |
| No | Ref | | | |
| Yes | 1.08(0.97,1.22) | 0.158 | 1.15(1.04,1.27) | 0.004 |
| Chronic pulmonary disease |  |  |  |  |
| No | Ref | | | |
| Yes | 1.13(1.00,1.28) | 0.048 | 1.23(1.11,1.36) | <0.001 |
| Diabetes |  |  |  |  |
| No | Ref | | | |
| Yes | 0.82(0.75,1.01) | 0.061 | 0.91(0.81,1.02) | 0.103 |
| Renal disease |  |  |  |  |
| No | Ref | | | |
| Yes | 0.76(0.63,0.91) | 0.004 | 0.80(0.68,0.93) | 0.004 |
| Malignant cancer |  |  |  |  |
| No | Ref | | | |
| Yes | 1.17(1.03,1.33) | 0.014 | 1.26(1.14,1.40) | <0.001 |
| Liver disease |  |  |  |  |
| No | Ref | | | |
| Yes | 1.62(1.34,1.96) | <0.001 | 1.94(1.64,2.29) | <0.001 |
| Metastatic solid tumor |  |  |  |  |
| No | Ref | | | |
| Yes | 1.46(1.12,1.90) | 0.004 | 1.64(1.31,2.05) | <0.001 |
| High HCT - ALB |  |  |  |  |
| < 6.7 | Ref | | | |
| ≥6.7 | 1.50(1.36,1.65) | <0.001 | 1.71(1.58,1.87) | <0.001 |

### Additional file 1: Table S2 The result of multivariate regressions of eICU-CRD dataset

|  | ICU Mortality P value | | Hospital Mortality P value | |
| --- | --- | --- | --- | --- |
| Variable | OR(95%CI) | P value | OR(95%CI) | P value |
| Age | 1.03(1.01,1.04) | <0.001 | 1.02(1.01,1.03) | <0.001 |
| SOFA | 1.20(1.16,1.24) | <0.001 | 1.18(1.14,1.21) | <0.001 |
| Gender |  |  |  |  |
| Male | Ref | | | |
| Female | 1.11(0.91,1.36) | 0.292 | 1.27(1.07,1.50) | 0.007 |
| BMI |  |  |  |  |
| Underweight | Ref | | | |
| Normalweight | 0.80(0.51,1.26) | 0.321 | 0.93(0.63,1.37) | 0.696 |
| Overweight | 0.88(0.56,1.39) | 0.570 | 0.85(0.58,1.26) | 0.412 |
| Obese | 0.62(0.40,0.98) | 0.036 | 0.71(0.48,1.05) | 0.085 |
| Ventilation |  |  |  |  |
| No | Ref | | | |
| Yes | 2.45(1.55,4.05) | <0.001 | 1.86(1.36,2.58) | <0.001 |
| Dialysis |  |  |  |  |
| No | Ref | | | |
| Yes | 2.88(2.17,3.82) | <0.001 | 2.32(1.77,3.05) | <0.001 |
| Vasopressor |  |  |  |  |
| No | Ref | | | |
| Yes | 2.49(1.91,3.27) | <0.001 | 1.13(0.91,1.38) | 0.258 |
| Congestive heart Failure |  |  |  |  |
| No | Ref | | | |
| Yes | 1.26(1.03,1.56) | 0.027 | 1.19(0.99,1.42) | 0.059 |
| Chronic pulmonary disease |  |  |  |  |
| No | Ref | | | |
| Yes | 1.28(1.04,1.58) | 0.021 | 1.06(0.89,1.28) | 0.473 |
| Diabetes |  |  |  |  |
| No | Ref | | | |
| Yes | 1.06(0.86,1.31) | 0.565 | 1.06(0.88,1.26) | 0.544 |
| Renal disease |  |  |  |  |
| No | Ref | | | |
| Yes | 0.80(0.46,0.10) | 0.048 | 1.00(0.83,1.20) | 0.998 |
| Malignant cancer |  |  |  |  |
| No | Ref | | | |
| Yes | 1.17(0.89,1.53) | 0.267 | 1.52(1.21,1.90) | <0.001 |
| Liver disease |  |  |  |  |
| No | Ref | | | |
| Yes | 1.36(1.05,1.75) | 0.025 | 1.65(1.31,2.07) | <0.001 |
| Metastatic solid tumor |  |  |  |  |
| No | Ref | | | |
| Yes | 2.50(1.75,3.59) | <0.001 | 2.06(1.51,2.82) | <0.001 |
| High HCT - ALB |  |  |  |  |
| < 6.7 | Ref | | | |
| ≥6.7 | 1.41(1.15,1.72) | 0.001 | 1.27(1.07,1.51) | 0.007 |

### Additional file 1: Table S3 The result of multivariate regressions of MIMIC-IV dataset
